# Effectiveness of Trastuzumab Combined With Capecitabine Treatment in a Patient With Hilar Cholangiocarcinoma Complicated by Liver Metastases With an *ERBB2*-Activating Mutation: A Case Report

**DOI:** 10.3389/fonc.2022.918297

**Published:** 2022-07-07

**Authors:** Daobing Zeng, Xiaofei Zhao, Liang Di, Luyan Lou, Yanfang Song, Yanrui Zhang, Huanhuan Liu, Guangming Li

**Affiliations:** ^1^ General Surgery Department, Beijing Youan Hospital, Capital Medical University, Beijing, China; ^2^ Clinical Center for Liver Cancer, Capital Medical University, Beijing, China; ^3^ Medical Affairs Department, Acornmed Biotechnology Co. Ltd., Beijing, China

**Keywords:** cholangiocarcinoma, *ERBB2* mutation, treatment, trastuzumab, case report

## Abstract

The identification of *ERBB2* (*HER2*) alteration in some solid tumors has become critically important due to the actionable events predictive of response to anti-HER2 therapy. However, the efficacy of *ERBB2* mutated hilar cholangiocarcinoma (hCCA) against ERBB2 is rarely reported. Here we report a 76-year-old female diagnosed with hCCA complicated by liver metastases after radical resection. The next-generation sequencing assay showed that the tumor had an *ERBB2* mutation. Then, the patient was treated with trastuzumab plus capecitabine. After 2 months of treatment, she had a partial response. Until now, the patient is still alive. This study has shown the potential of trastuzumab combined with capecitabine as an effective treatment for hilar cholangiocarcinoma complicated by liver metastases harboring *ERBB2* alterations.

## Introduction

Cholangiocarcinoma (CCA) is a heterogeneous malignancy that can occur at every point in the biliary tree, from the canals of Hering to the main bile duct (1){Banales, 2016 #3}{Banales, 2016 #3}. CCAs are classified into intrahepatic (iCCA) and extrahepatic (eCCA) categories on the basis of anatomical origin (2). The eCCA can be further separated into two subtypes: the proximal or hilar (HCCA) accounts for 60–70% and the distal for 20–30% of CCA (3).

hCCA is a common malignant tumor with a relatively poor prognosis. The overall 5-year survival rate is approximately 13–40% (4). The gold standard of treatment for patients with unresectable hilar cholangiocarcinoma is palliative chemotherapy. However, there was a poor outcome with radiation and systemic chemotherapy, such as with gemcitabine and cisplatin (5). Thus, a novel therapeutic approach is warranted.

Targeted therapies are rapidly transforming the therapeutic paradigm for biliary tract cancer, particularly for iCCA, where targeting *FGFR2* fusions, *IDH 1/2* mutations, and *BRAF* V600E mutations are becoming a reality (6, 7). Unlike iCCA, targeted therapies for hCCA are rare.

Herein we report a rare case of a female patient with *ERBB2*-mutant hCCA who responds well to the treatment of trastuzumab in combination with capecitabine.

## Case Presentation

A 76-year-old woman was admitted to a local hospital after she experienced itchy skin plus dark yellow urine for several days. The patient was diagnosed with Bismuth type IV hCCA after imageological diagnosis and received percutaneous transhepatic cholangiodrainage (PTCD) on February 5, 2021. The total bilirubin concentration was 400 µmol/L before PTCD, and the daily bile drainage was approximately 300-400 ml. The preoperative total bilirubin level was reduced to the normal level of 19.6 µmol/L. Then, she visited Beijing Youan Hospital, Capital Medical University, for further operative treatment. Enhanced CT of the upper abdomen and magnetic resonance cholangiopancreatography indicated high biliary obstruction and a high possibility of hilar cholangiocarcinoma, involving the upper to the middle common bile duct, and lesions after PTCD. On March 17, 2021, the patient underwent hilar radical resection, and the resection segments were reconstructed by “basin-shape” hepaticojejunostomy. The clinical data were collected and analyzed. A postoperative pathological examination revealed adenocarcinoma with lymph node metastasis. The patient fell accidentally after discharge from the hospital and experienced a fracture of the left femoral neck. Therefore, she did not undergo postoperative treatment until she came to the hospital for review in September 2021. Abdominal contrast-enhanced CT revealed a low-density mass in the left medial hepatic lobe of the liver. After receiving multidisciplinary treatment, the patient was considered unsuitable for surgical treatment, ablation therapy, and radiotherapy.

With the patient’s consent, the tissue sample obtained during surgery was subjected to next-generation sequencing (NGS) using an 808-gene panel in a College of American Pathologists (CAP)-certified lab. Somatic gene mutations have been detected in the tissue, including *ERBB2* R678Q, *PTCH1* c.747-2A>T, *TP53* E286Q, *ARID1A* Q2176X, *SMAD4* D493Y, and *SMAD4* R361H (**Table 1**). The NGS results indicated low-level microsatellite instability and low tumor mutational burden (5.58 mutants/Mb).

**Table 1:** Summary of gene test results and mutations that may have clinical significance.

Gene	Exon	Mutation	Variation frequency
*ERBB2*	Exon 17	R678Q	41.60%
*PTCH1*	Exon 6	c.747-2A>T	33.25%
*TP53*	Exon 8	E286Q	27.05%
*ARID1A*	Exon 20	Q2176X	25.78%
*SMAD4*	Exon 12	D493Y	26.71%
*SMAD4*	Exon 9	R361H	3.38%

**Figure 1:**
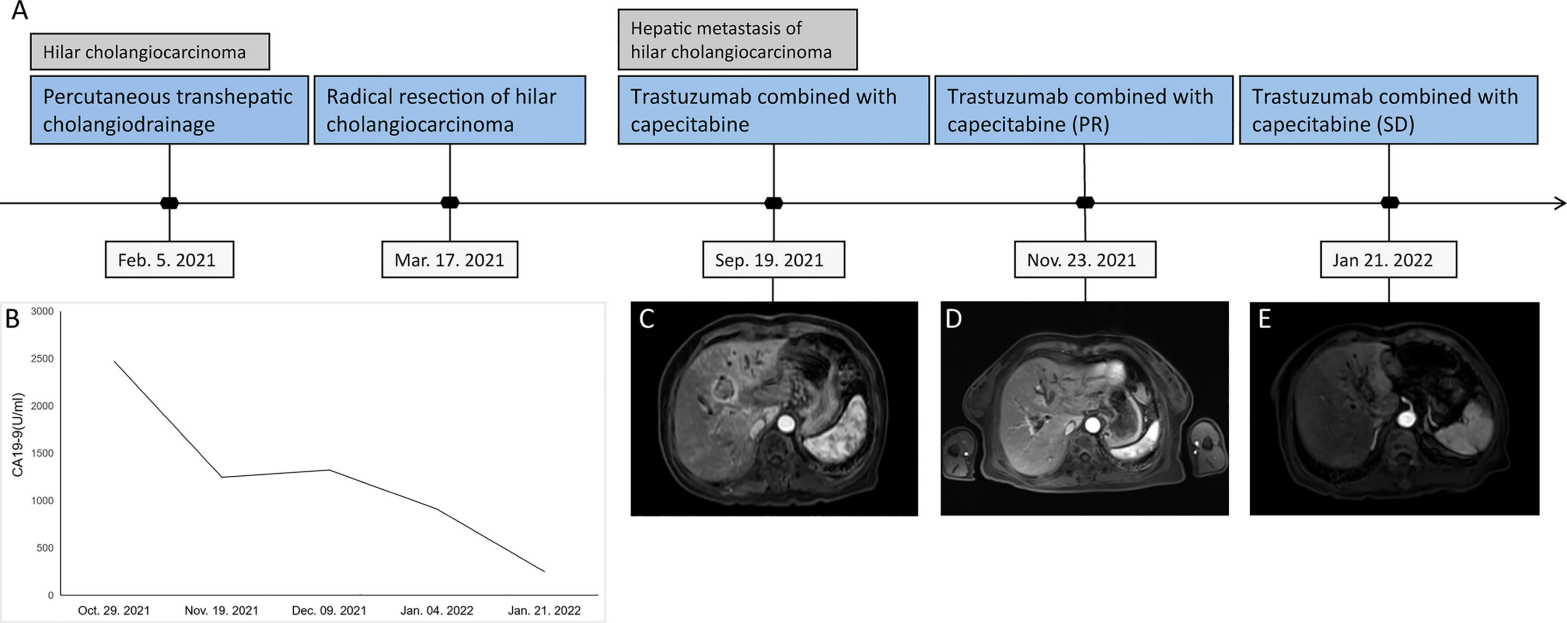
Tumor progression of the patient with postoperative liver metastases from hilar cholangiocarcinoma. **(A)** The timeline of therapies and tumor progression are indicated. **(B)** Line chart showing changes in the levels of the tumor marker CA 19-9 during the course of treatment. **(C–E)** MR scan of the lesion in the bile duct before and after 2 and 4 months of capecitabine plus trastuzumab treatment. MR, magnetic resonance; CA 19-9, cancer antigen 19-9.

With the patient’s informed consent, we considered trastuzumab combined with capecitabine as the first-line treatment strategy. The initial loading dose of trastuzumab was 420 mg, followed by 380 mg at 3 weeks later, concomitant with capecitabine 2,000 mg/m^2^ for 14 days in 21-day cycles. Tumor response is “objectified” according to the response evaluation criteria in solid tumors, whereby the longest diameter of the target lesion that can be best visualized is monitored over time—depending on the clinical situation, *e*.*g*., every 2 months. After 2 months, the MR scan showed that the volume of some lesions was reduced by 51.9%, from 27 to 13 mm, indicating that the patient had a partial response (PR). We then continued to treat the patient with trastuzumab combined with capecitabine. After another 2 months, the lesion diameter was still 13 mm, which was considered a stable disease (SD), and no metastatic lesions in other organs were found. In addition, the levels of serum tumor markers were significantly reduced during the treatment (**Figure 1**). At the time of this report, the patient was alive.

## Discussion

Advances in genomic research have promoted the development of personalized medicine for the treatment of cancer patients. Here we reported an hCCA patient harboring the *ERBB2* R678Q mutation who acquired a response to trastuzumab and capecitabine treatment. To our knowledge, this is the first report to claim that *ERBB2* mutations can be used as trastuzumab targets in hCCA.

ERBB2-targeted therapy has achieved remarkable success in the treatment of metastatic breast cancer and gastric cancer. Similarly, recent studies have identified that patients with ERBB2 overexpression or amplification could potentially benefit from ERBB2-targeted therapy in biliary tract cancer (trastuzumab, not lapatinib, has therapeutic effects on Chinese patients with HER2-positive cholangiocarcinoma) (8){Law, 2012 #21;Law, 2012 #21}.

Jeong reported that a biliary tract cancer patient with HER2 overexpression received trastuzumab-pkrb, gemcitabine, and cisplatin and showed an overall response rate of 50.0% (including SD and PR) (9). Yarlagadda *et al.* demonstrated that a CCA patient with *ERBB2* amplification was treated with dual HER2-directed therapy (trastuzumab/pertuzumab) and responded very well with regression of tumor on imaging (10). Consistent with this result, our study showed that an hCCA patient with *ERBB2* mutated can benefit from capecitabine combined with trastuzumab treatment. This may implicate that ERBB2-targeted therapy could be a favorable option in hCCA with *ERBB2* variation.

ERBB receptors contain an extracellular domain (ECD), a transmembrane domain (TMD), an intracellular region that consists of a juxtamembrane domain (JMD), a kinase domain, and a carboxy terminal tail domain (11). Several studies reported that TMD variation and ECD of *ERBB2* may stabilize ERBB2 heterodimerization with other EGFR family members and favor a kinase-active conformation (12). TMD variations are located within the glycine zipper motif at the N‐terminal portion of TMD, which is critically important to the dimerization of ERBB2 to other EGFR family members (12).

Trastuzumab is a monoclonal antibody specifically designed to target the ERBB2. The mechanisms of action of trastuzumab include antibody-dependent cellular cytotoxicity, antibody-mediated ERBB2 internalization followed by receptor degradation, and inhibition of ERBB2 dimerization (13). *ERBB2* amplification with corresponding HER2 protein overexpression is associated with sensitivity to therapies targeting HER2, including lapatinib, pertuzumab, and trastuzumab, which were approved by the US Food and Drug Administration. Recent evidence has demonstrated that the presence of *ERBB2* mutation can predict clinical responses to anti-HER2-targeted therapies (14).

The recent results from the SUMMIT study have shown that the clinical benefit from ERBB2-targeted therapies (neratinib) may be dependent on the type of *ERBB2* alteration and type of tumor (15). Mou *et al.* reported that a CCA patient with *ERBB2* gene S310F in the ECD received trastuzumab combined with chemotherapy and showed therapeutic effects, decreased tumor burden, and improved quality of life (16). Herein we found an hCCA patient, with Pahuja et al. indicating that the *ERBB2* R678Q mutation located at the JMD might have a significant effect on TMD geometry and dimerization (17). Therefore, we speculated that the JMD mutant R678Q might respond to trastuzumab.

## Concluding Remarks

In conclusion, we were able to identify *ERBB2* JMD mutations on NGS in a patient with hCCA and found anti-HER2 therapy as an effective treatment strategy. This will have implications for other patients with hCCA in terms of the identification of this target and considerations toward anti-HER2 therapy on or off trial.

## Data Availability Statement

The original contributions presented in the study are included in the article/supplementary materials. Further inquiries can be directed to the corresponding author.

## Ethics Statement

Written informed consent was obtained from the individual(s) for the publication of any potentially identifiable images or data included in this article.

## Author Contributions

XZ and LD participated in collecting data. LL and YS drafted the manuscript. HL and YZ revised and commented on the draft. DZ and GL contributed to the scientific review and final approval of this manuscript. All authors read and approved the final manuscript.

## Conflict of Interest

Author LL, YS, YZ, and HL were employed by the company Acornmed Biotechnology Co., Ltd.

The remaining authors declare that the research was conducted in the absence of any commercial or financial relationships that could be construed as a potential conflict of interest.

## Publisher’s Note

All claims expressed in this article are solely those of the authors and do not necessarily represent those of their affiliated organizations, or those of the publisher, the editors and the reviewers. Any product that may be evaluated in this article, or claim that may be made by its manufacturer, is not guaranteed or endorsed by the publisher.
